# Clearance of viable *Mycobacterium ulcerans* from Buruli ulcer lesions during antibiotic treatment as determined by combined 16S rRNA reverse transcriptase /IS 2404 qPCR assay

**DOI:** 10.1371/journal.pntd.0005695

**Published:** 2017-07-03

**Authors:** Mabel Sarpong-Duah, Michael Frimpong, Marcus Beissner, Malkin Saar, Ken Laing, Francisca Sarpong, Aloysius Dzigbordi Loglo, Kabiru Mohammed Abass, Margaret Frempong, Fred Stephen Sarfo, Gisela Bretzel, Mark Wansbrough-Jones, Richard Odame Phillips

**Affiliations:** 1 Kwame Nkrumah University of Science and Technology (KNUST), School of Medical Sciences and Kumasi Centre for Collaborative Research in Tropical Medicine (KCCR), Kumasi, Ghana; 2 Department of Infectious Diseases and Tropical Medicine (DITM), University Hospital, Ludwig-Maximilians-University, Munich, Germany; 3 Institute of Infection and Immunity, St George's University of London, London, United Kingdom; 4 Agogo Presbyterian Hospital, Agogo, Ghana; Johns Hopkins Bloomberg School of Public Health, UNITED STATES

## Abstract

**Introduction:**

Buruli ulcer (BU) caused by *Mycobacterium ulcerans* is effectively treated with rifampicin and streptomycin for 8 weeks but some lesions take several months to heal. We have shown previously that some slowly healing lesions contain mycolactone suggesting continuing infection after antibiotic therapy. Now we have determined how rapidly combined *M*. *ulcerans* 16S rRNA reverse transcriptase / IS*2404* qPCR assay (16S rRNA) became negative during antibiotic treatment and investigated its influence on healing.

**Methods:**

Fine needle aspirates and swab samples were obtained for culture, acid fast bacilli (AFB) and detection of *M*. *ulcerans* 16S rRNA and IS*2404* by qPCR (16S rRNA) from patients with IS*2404* PCR confirmed BU at baseline, during antibiotic and after treatment. Patients were followed up at 2 weekly intervals to determine the rate of healing. The Kaplan-Meier survival analysis was used to analyse the time to clearance of *M*. *ulcerans* 16S rRNA and the influence of persistent *M ulcerans* 16S rRNA on time to healing. The Mann Whitney test was used to compare the bacillary load at baseline in patients with or without viable organisms at week 4, and to analyse rate of healing at week 4 in relation to detection of viable organisms.

**Results:**

Out of 129 patients, 16S rRNA was detected in 65% of lesions at baseline. The *M*. *ulcerans* 16S rRNA remained positive in 78% of patients with unhealed lesions at 4 weeks, 52% at 8 weeks, 23% at 12 weeks and 10% at week 16. The median time to clearance of *M*. *ulcerans* 16S rRNA was 12 weeks. BU lesions with positive 16S rRNA after antibiotic treatment had significantly higher bacterial load at baseline, longer healing time and lower healing rate at week 4 compared with those in which 16S rRNA was not detected at baseline or had become undetectable by week 4.

**Conclusions:**

Current antibiotic therapy for BU is highly successful in most patients but it may be possible to abbreviate treatment to 4 weeks in patients with a low initial bacterial load. On the other hand persistent infection contributes to slow healing in patients with a high bacterial load at baseline, some of whom may need antibiotic treatment extended beyond 8 weeks. Bacterial load was estimated from a single sample taken at baseline. A better estimate could be made by taking multiple samples or biopsies but this was not ethically acceptable.

## Author summary

Buruli ulcer (BU) caused by *Mycobacterium ulcerans* is effectively treated with rifampicin and streptomycin for 8 weeks but some lesions take several months to heal. We have shown previously that some slowly healing lesions contain the *M*. *ulcerans* toxin, mycolactone, suggesting continuing infection after completion of antibiotic therapy. In the present study we have determined how soon *M*. *ulcerans* was killed during antibiotic treatment using the *M*. *ulcerans* 16S rRNA assay combined with qPCR for IS*2404* to detect live bacilli in clinical samples and investigated its influence on healing. This assay is more sensitive than culture for the organism. Using samples collected from one hundred and twenty-nine BU patients prior to antibiotic treatment, viable organisms were detected by culture in 34% but the 16S rRNA assay was positive in 65%. The 16S rRNA remained positive in 78% of patients with unhealed lesions at 4 weeks, 52% at 8 weeks, 23% at 12 weeks, and 10% at week 16. Lesions with positive 16S rRNA after antibiotic treatment also contained a higher number of bacteria at baseline, had a lower rate of healing at week 4 and took a longer time to heal compared with those in which the organism was undetectable at baseline or by week 4. Positive 16S rRNA was less likely in ulcerative compared with nodular forms of disease 4 weeks after antibiotic treatment. It may be possible to shorten the treatment to 4 weeks in patients with low numbers of bacteria at baseline. Since persistent infection appears to contribute to slow healing, some patients with a high bacterial load at baseline may need antibiotic treatment for longer than 8 weeks.

## Introduction

Buruli ulcer is a neglected tropical disease caused by infection with *Mycobacterium ulcerans* (Mu) which is common in rural parts of West African countries including Ghana [1]. It causes large, disfiguring skin ulcers mainly in children aged 5 to 15 years although any age can be affected [2]. The initial lesion is a subcutaneous painless nodule tethered to the skin or an intradermal plaque sometimes associated with oedema. These enlarge over a period of days to weeks and ulcerate in the centre. Ulcers are painless and have a necrotic base and irregular, undermined edges. There is surrounding oedema in about 10% of cases. Ulcers enlarge progressively and may cover the whole of a limb or the trunk if left untreated but the patient remains systemically well unless secondary bacterial infection occurs [3] [4] [5]. The mode of transmission remains unknown[5, 6]but there have been major advances in understanding the mechanism of disease since the establishment of the WHO Buruli ulcer initiative in 1998 together with improved diagnosis and clinical management.

Treatment of Buruli ulcer has changed considerably since 2004 with the introduction of antibiotics as an alternative to surgery. It has now been established that the combination of rifampicin and streptomycin administered daily for 8 weeks is effective in healing all forms of lesion caused by Mu disease and this has reduced the recurrence rate from 6–47% after surgery to 0–2% after antibiotic treatment [6, 7]. This treatment can be administered by community health nurses and admission to hospital is rarely necessary except when skin grafting is needed. The current duration of antibiotic therapy (8 weeks) was based on observations in patients with early Mu lesions which were excised after treatment for 2, 4, 8 or 12 weeks. All lesions remained culture positive after 2 weeks but thereafter all were culture negative [3]. Thus it is likely that a shorter course of treatment may be successful in some patients which would be highly desirable, not least because streptomycin has to be injected intramuscularly. This is supported by recent experience of treating *M ulcerans* disease in Australia with antibiotic durations of less than 8 weeks suggesting that successful outcomes may be achieved in selected patients [8]. In spite of the success of rifampicin and streptomycin treatment for 8 weeks some lesions take much longer than others to heal despite having appeared identical before treatment. Available data from various studies suggest that healing of up to two thirds of patients occurs within 25 weeks from the start of treatment [9–11].

One reason for slow healing may be that active infection persists despite antibiotic treatment for 8 weeks. In our recent study of BU treated with rifampicin and streptomycin for 8 weeks, persistent infection with *M*. *ulcerans* was shown by positive cultures in some lesions 4 weeks after completion of antibiotic treatment despite full adherence to therapy. Furthermore mycolactone, the toxin produced by *M*. *ulcerans*, was detected in lesions which were culture negative as well as in culture positive samples, suggesting that it is a more sensitive marker for the presence of viable organisms [12]. However it is not known how long mycolactone can remain in human BU lesions after *M*. *ulcerans* is killed and it is vital to establish how often infection persists after a standard course of antibiotic treatment.

Reverse transcriptase assays targeting ribosomal or messenger RNA have been applied successfully for the rapid detection of viable mycobacteria in clinical samples from patients with tuberculosis, leprosy and recently Buruli ulcer [13] [14] [15] and as a surrogate for response to chemotherapy in tuberculosis [13]. With respect to Buruli ulcer, the assay is fast, 100% specific for *M*. *ulcerans* and highly sensitive with an analytical sensitivity of 6 templates of the targeted 16S rRNA. The excellent performance on clinical samples makes this tool highly promising for monitoring the therapeutic response with the goal of optimizing the duration of antimycobacterial treatment [15]. The aim of the present study was to determine how rapidly combined *M*. *ulcerans* 16S rRNA reverse transcriptase / IS*2404* qPCR assay (hereafter referred to as 16S rRNA) became negative during antibiotic treatment and to relate this to the rate of healing.

## Materials and methods

### Patients

In the period from June 2013 to June 2015, patients more than 5 years of age with suspected Buruli ulcer and subsequent confirmation by *M*. *ulcerans* IS*2404* dry reagent based (DRB) PCR presenting to treatment clinics at the Tepa Government Hospital, Nkawie-Toase Government Hospital, Dunkwa Government Hospital and Agogo Presbyterian Hospital were screened for inclusion. Patients who had already been under antimycobacterial treatment at the time of study initiation were excluded.

### Study procedures

Demographic data were collected using standard BU01 forms from the WHO together with a careful history to establish when lesions were first observed and their type. The dimensions of lesions were documented with Silhouette (ARANZ Medical, Christchurch, New Zealand) a 3-dimensional imaging and documentation system together with digital photographs. The Silhouette camera captures an image of the wound, a tracing of the wound boundary is generated and the wound dimensions including the area, depth and volume are automatically calculated. For oedematous lesions, only digital photographs were obtained. Patients were reviewed at 2 weekly intervals during standard antibiotic treatment and monthly thereafter with further recordings of clinical data as routinely conducted for all BU patients until complete healing. These measurements enabled calculation of healing rate at week 4 and predicted healing time in relation to lesion size and type. Rate of healing in mm per week was calculated by subtracting the mean diameter of the lesion in millimeters determined at week 4 from that determined at week 0 and dividing this result by 4. Mean diameter was the mean of the maximum diameter and the largest diameter at right angles to that [16]. Two fine needle aspirates (FNA) or swabs samples were collected from skin lesions to confirm the diagnosis of Buruli ulcer by microscopy and conventional IS*2404* DRB PCR. An additional sample for culture and another for the 16S rRNA reverse transcriptase/IS*2404* qPCR assay (16S rRNA) were collected at baseline and during (week 4 and 8) or after treatment (week 12 and 16) from unhealed lesions, immediately placed in either 500μl PANTA media or 500μl RNA protect respectively on site. Human GAPDH mRNA assay was performed on the samples in the RNA protect to assess the stability of the RNA in the solution (Qiagen, UK).

All routine laboratory tests were conducted at Kumasi Centre for Collaborative Research in Tropical Medicine (KCCR) immediately upon arrival of samples. Prior to the study a human GAPDH mRNA reverse transcriptase qPCR was established and validated at the Department for Infectious Diseases and Tropical Medicine (DITM) of the University Hospital of the Ludwig-Maximilians-University (LMU) in Munich, Germany. During the study all molecular assays were conducted at the KCCR by trained laboratory staff supervised by Kwame Nkrumah University of Science and Technology (KNUST) staff.

Whole genome DNA and whole transcriptome RNA were extracted at the KCCR immediately on arrival of samples in RNA protect and subjected to the *M*. *ulcerans* 16S rRNA assay [15].

### Routine laboratory confirmation

For laboratory confirmation of Buruli ulcer disease, AFB microscopy, IS*2404* dry reagent based (DRB)-PCR and cultures were performed. IS*2404* qPCR were performed by well established methods as previously described [17][18] [15]. IS*2404* qPCR was also performed on all samples. A final diagnosis of Buruli ulcer was based on IS*2404* DRB-PCR and qPCR results which were the most sensitive tests.

### Combined 16S rRNA reverse transcriptase / IS*2404* qPCR assay

FNA and swab samples were transported from study site to the KCCR stabilized in 500 μl RNA protect (Qiagen, UK). Whole transcriptome RNA and whole genome DNA were extracted from the same clinical sample. The RNA and DNA isolation was carried out within 5 hours of sample collection using the AllPrep DNA/RNA Micro kit (Qiagen, UK) as previously described with minor modification[15]. Here, homogenizing was carried out with the QiaShredder (Qiagen, UK) according to the manufacturers instruction in a biosafety cabinet. 12 μl RNA extracts were immediately reverse transcribed whilst 50 μl DNA extracts obtained were stored at 4–8°C (short-term) or -20°C (long-term).

To remove potentially contaminating genomic DNA (gDNA) from the *M*. *ulcerans* whole transcriptome RNA extracted, 2 μl DNA wipe out buffer (Qiagen, UK) was added to 12 μl of the total RNA extracts, incubated for 5 min at 42°C and the reaction was terminated by incubating at 95°C for 3 min. 2 μl gDNA free *M*. *ulcerans* whole transcriptome RNA extracted was included as a wipe out control. The remaining *M*. *ulcerans* whole transcriptome RNA was then reverse transcribed into cDNA using QuantiTect Reverse transcription kit (Qiagen, UK) according to the manufacturer’s instructions as described elsewhere[15]. The cDNA samples were stored at -20°C until further processing.

“To exclude false negative 16S rRNA RT qPCR results (e.g. due to RNA degradation during sample transport or RNA extraction procedures), the cDNA prepared as described above was subjected to qPCR for detection of the human glyceraldehyde-3-phosphate dehydrogenase (GAPDH) mRNA (S1 Protocol)[18]. The performance of the GAPDH mRNA reverse transcriptase qPCR is provided as supplementary material (S2 Protocol). All whole transcriptome RNA extracts from Buruli ulcer patients tested positive when subjected to GAPDH mRNA RT qPCR at baseline.

The cDNA was then subjected to 16S rRNA qPCR and DNA to IS*2404* qPCR to increase the specificity for *M*. *ulcerans* and quantification of the bacterial load as previously described [15]. Quantitative PCR of IS*2404* (DNA), and 16S rRNA (cDNA) targets were carried out at 95°C for 15 min, and then 40 cycles of 95°C for 15 sec and 60°C for 60 sec in a BioRad CFX 96 real time PCR detection system (BIORAD, Singapore). Each run included negative extraction controls, negative “no template” controls, negative gDNA wipe-out controls (16S rRNA qPCR only), inhibition controls (exogenous IPC) and positive controls. Ten fold serial dilutions of known amounts of a plasmid standard of IS*2404* (99 bp) and 16S rRNA (147 bp) (Eurofins MWG Operon, Ebersberg, Germany) were included with PCR amplification for preparation of a standard curve. *M*. *ulcerans* bacillary loads in original clinical samples were calculated based on threshold cycle values per template of IS*2404* qPCR (standard curve method) adjusted to the whole amount of DNA extract and the known copy number of 207 IS*2404* copies per *M*. *ulcerans* genome on average.

### Statistical analysis

The raw data generated from the study was entered in Microsoft Excel (Microsoft Corporation, Redmond, WA) and analyzed using Graphpad Prism version 5.0 (GraphPad Software, Inc., La Jolla, CA) and Microsoft Excel (Microsoft Corporation). The Kaplan-Meier survival analysis (log rank test) was used to determine the time to clearance of *M*. *ulcerans* 16S rRNA, as well as to determine the influence of persistent *M ulcerans* 16S rRNA on time to healing. This approach was used to offset bias due to patient censoring for not showing up at study time points. Mann Whitney test was used to compare the bacillary load at baseline in patients with presence or absence of viable organisms at week 4, and also to analyse rate of healing at week 4 in relation to detection of viable organisms. Mann Whitney test were used due to variable distribution of data. Fisher’s exact test was used to compare positive results of 16S rRNA assay with culture due to small sample size. P value < 0.05 was considered statistically significant in all the analyses. All statistical tests were two-tailed.

### Ethics statement

Verbal and written informed consent was obtained from all eligible participants, and from parents or legal representatives of participants aged 18 years or younger. Ethical approval was obtained from the Committee of Human Research Publication and Ethics, School of Medical Sciences, Kwame Nkrumah University of Science and Technology, Kumasi, Ghana (CHRPE/AP/229/12).

## Results

### Characteristics and diagnosis of study participants

Of 150 patients presenting to treatment centers with clinically suspected Buruli ulcer, *M*. *ulcerans* infection was confirmed by IS*2404* PCR in 129 cases (Table 1): in 104 out of these by gel-based DRB PCR and qPCR, and for the remaining 25 cases by IS*2404* qPCR only. Fifty seven (44%) had pre-ulcerative lesions and 16 (12%) had lesions larger than 15 cm in maximum diameter (category III). There were 8 lesions with oedema, 4 of which were pre-ulcerative. Out of 129 IS*2404* PCR positive patients, direct smears for the detection of AFB were available for 125 patients (96.9%) and 50 (40%) tested positive. Samples were taken for culture from 129 patients of which 44 (34%) were positive.

**Table 1:** Demographic data and diagnostic test results for Buruli ulcer patients.

	No. of Participants: N (%) n = 129
**Age(years)**	
Median(IQR ^e^)	14(10–30)
**Sex**	
Male Female	61 (47.3) 68 (52.7)
**Lesion Form** ^a^	
Nodule	29 (22.4))
Plaque	24 (18.6))
Oedema	4 (3.1))
Ulcer	68(52.7)
Ulcer with oedema	4 (3.1)
**Category of lesion**	
I (< = 5cm)	57 (44.2)
II(5-15cm)	56 (43.4)
III(>15cm)	16(12.4)
**Sample type**	
FNA	63(48.8)
Swab	66(51.2)
**Diagnostic confirmation**	
Microscopy^b^	50/125(40.0)
Culture^d^	44/129(34.1)
IS *2404* DRB-PCR^c^	104/127(81.9)
IS *2404* qPCR	129/129(100)

^a^FNA samples were taken from 3 patients presenting with ulcers because they did not have undermined edges and from 1 patient presenting with ulcerated oedema.

Diagnostic tests used in the study were smear microscopy for AFB, culture for *M*. *ulcerans*, dry-reagent-based (DRB) IS*2404* PCR and IS*2404* real time PCR (qPCR).

^b^ Microscopy was not done for 4 participants

^c^ IS*2404* DRB-PCR was not done for 2 participants

^d^6 of these patients had a positive *M*. *ulcerans* culture result from a sample taken after 4 weeks of antibiotics treatment

^e^ IQR interquartile range

### Detection of *M*. *ulcerans* 16S rRNA and response to antibiotic treatment

Positive results for *M*. *ulcerans* 16S rRNA were obtained in 84 out of 129 patients (65%) at baseline (Table 2). Although the sensitivity of 16S rRNA was substantially higher than that for culture (34%), 2 of 38 samples yielding a positive culture had negative 16SrRNA, presumably as a result of sampling error.

**Table 2:** Sensitivity of *M*. *ulcerans* 16S rRNA assay compared with culture for Buruli ulcer patients at baseline.

	Mu Culture (No. of patients^a^) N = 124		
Mu 16S rRNA N = 124	Positive (n = 38)	Negative (n = 86)	Sensitivity (95% CI)
Positive (n = 84)	36	48	95(82–99)
Negative (n = 40)	2	38	

^a^All Patients were *M*. *ulcerans* IS*2404 qPCR* positive.

**Fig 1:**
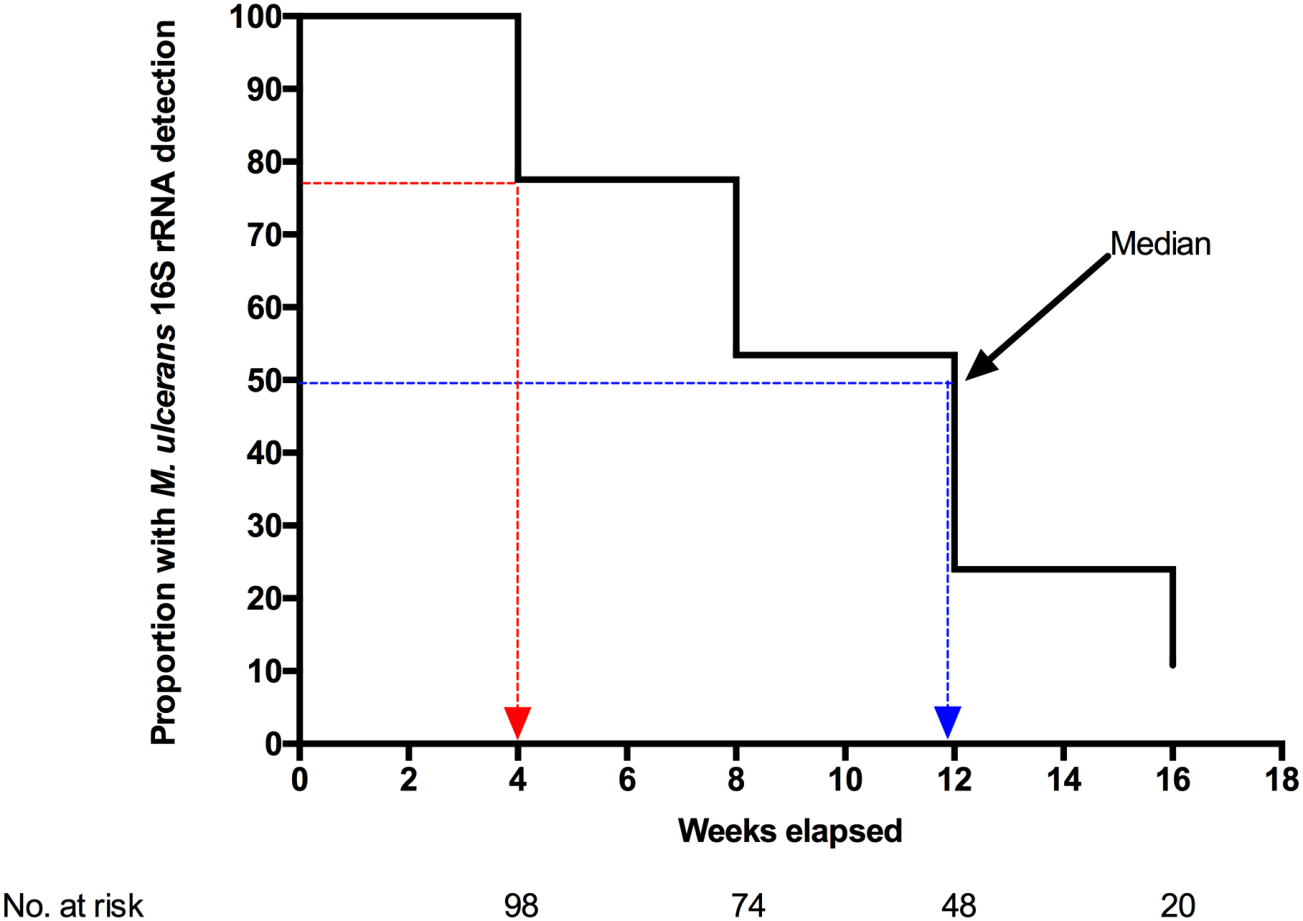
Kaplan-Meier analysis of *M*. *ulcerans* 16S rRNA in Buruli patients on antibiotic treatment. Blue line: Median time (weeks) for detection of *M*. *ulcerans* 16S rRNA. Red line: Proportion of patients with positive *M*. *ulcerans* 16S rRNA at week 4.

After initiation of antibiotic therapy, *M*. *ulcerans* 16S rRNA was detected in 78% of patients with unhealed lesions at 4 weeks, 52% at 8 weeks, 23% at 12 weeks, and 10% at week 16 (Fig 1). Of 15 patients censored at week 16 when sampling ended, 3 had positive *M*. *ulcerans* 16S rRNA but in 12 patients a sample could not be obtained. Thus despite antibiotic treatment for 8 weeks, positive 16S rRNA was still detected in 52% lesions sampled at week 8 and the median for detection of *M*. *ulcerans* by Kaplan-Meier curve analysis was 12 weeks (95% CI 8–16). The number of patients whose lesions yielded a positive *M*. *ulcerans* culture decreased to 24% at week 4, 5% at week 8 and none by week 16. *M*. *ulcerans* was detected by culture for a median time of 4 weeks (95% CI 4–6) (S1 Table).

### Relationship of bacterial load before treatment to clearance of *M*. *ulcerans* 16S rRNA

**Fig 2:**
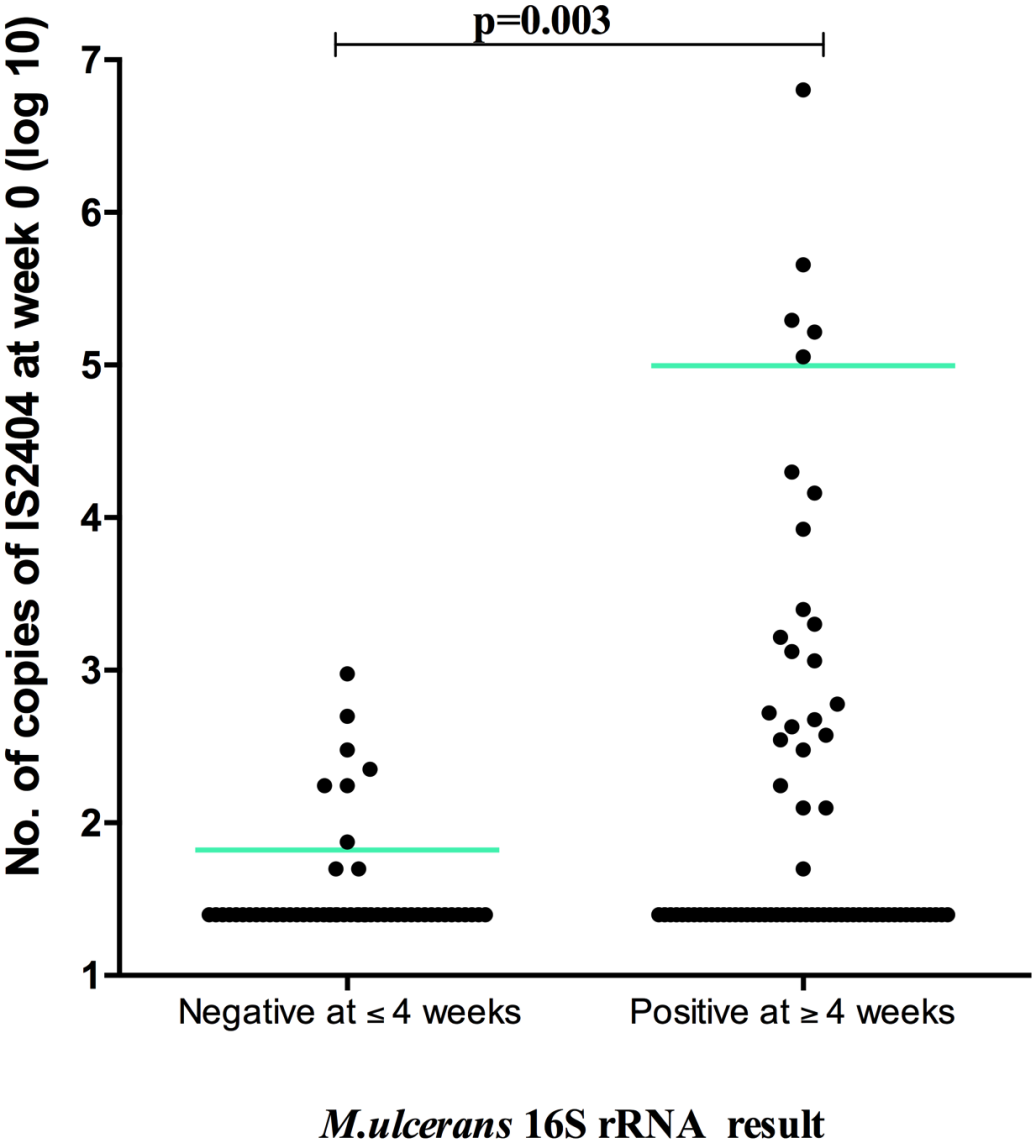
Comparison of baseline *M*. *ulcerans* IS*2404* in Buruli ulcer patients with a positive or negative 16S rRNA result at week 4.

Before antibiotic treatment, 28 patient lesions in which *M*. *ulcerans* 16S rRNA was negative and 27 patients with detectable *M*. *ulcerans* 16S rRNA at baseline but subsequently undetectable after 4 weeks of antibiotic treatment had a significantly lower bacterial load based on qPCR for IS*2404* (p = 0.003; Mann Whitney) (Fig 2), than those of 74 patients with detectable 16S rRNA at week 4 or later.

### Detection of *M*. *ulcerans* 16S rRNA and healing outcome

**Fig 3:**
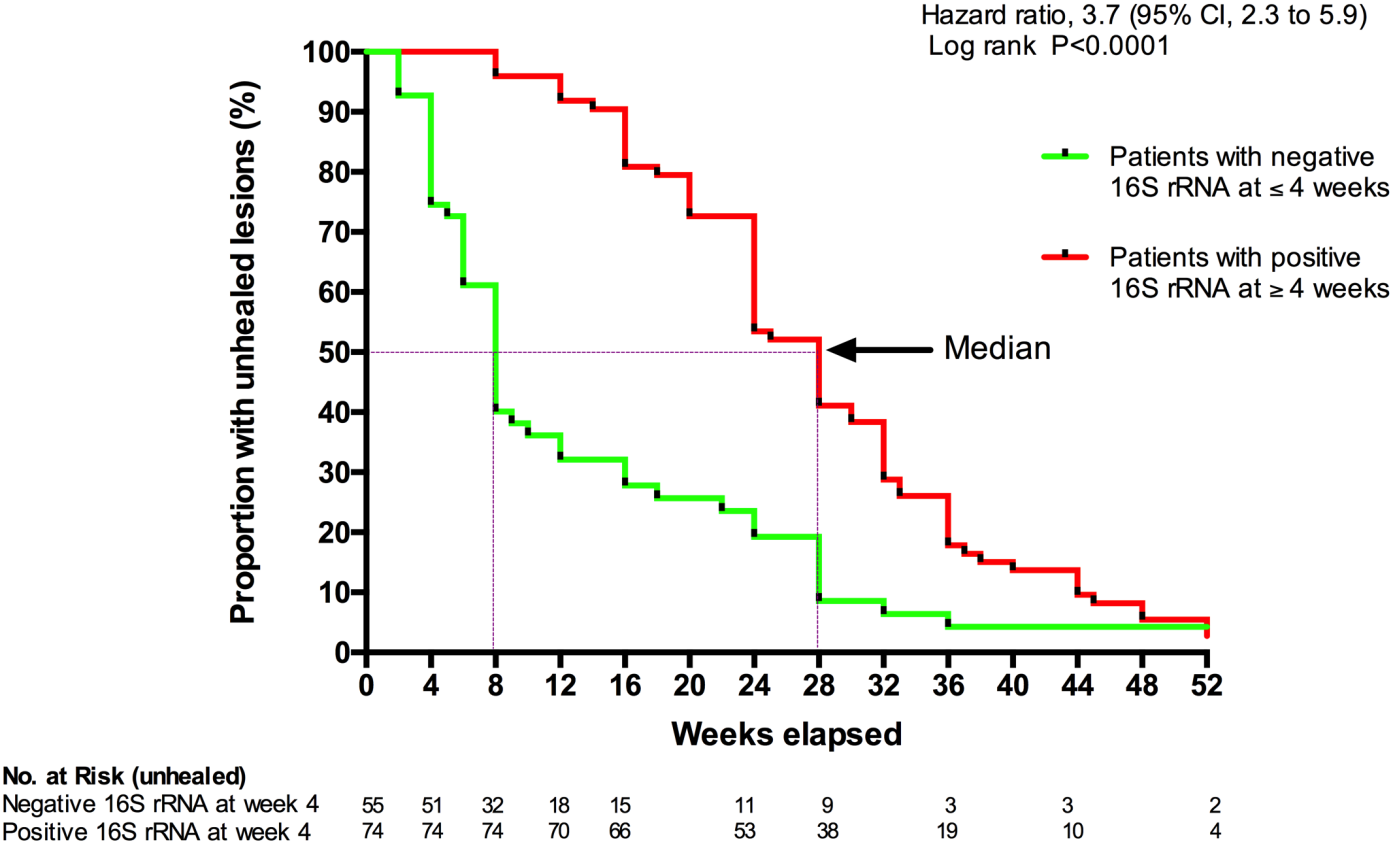
Survival curve for time to healing in Buruli patients with a negative or positive *M*. *ulcerans* 16S rRNA at week 4. Purple lines: Median time to healing.

**Fig 4:**
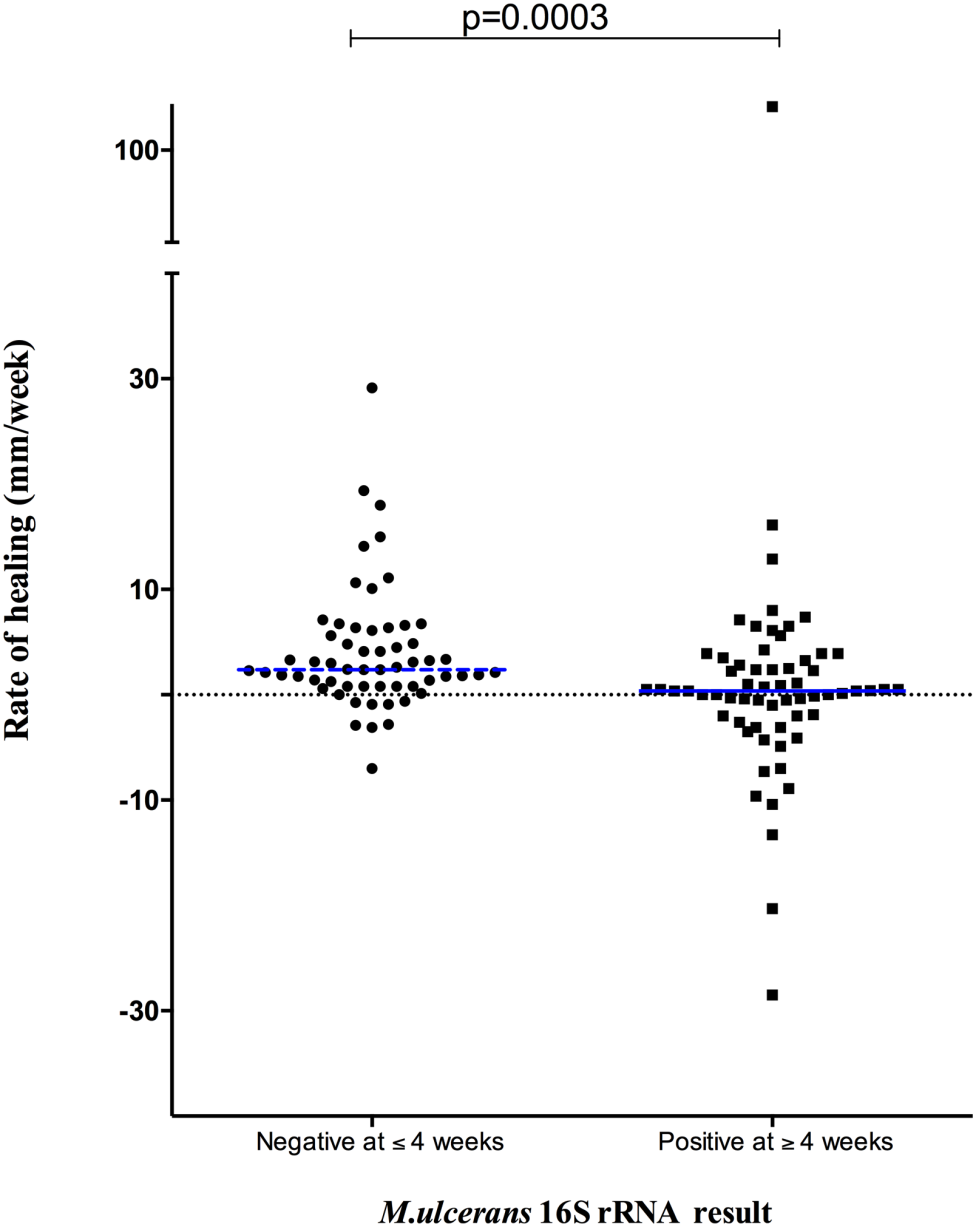
Rate of wound healing at week 4 in Buruli patients with a negative or positive *M*. *ulcerans* 16S rRNA. Rate of healing was highest in patients where *M*. *ulcerans* 16S rRNA was negative at baseline or 4 weeks after starting antibiotic treatment. The rate of healing at week 4 (ROH) was computed in millimeters per week by subtracting the mean diameter of the lesion at week 4 from that at week 0 and dividing this result by 4.

Patients with positive 16S rRNA at week 4 had a 3.7-fold increase (95% CI 2.43–5.04) in the time to complete healing of Buruli ulcer lesions compared to those with negative 16S rRNA result at week 4 (Fig 3). This was not attributable to lesion size at baseline because there was no significant difference in initial size of patient lesions with or without detectable 16S rRNA at week 4 (p = 0.0798, Mann Whitney). Fig 4 shows that the rate of wound healing (ROH) determined at week 4 was higher for patients with undetectable 16S rRNA at week 4 [2.4 (0.8 to 6.2) mm/week; median (interquartile range)] compared to those with positive 16S rRNA at week 4 [0.3 (-2.0 to 3.3) mm/week] (p = 0.0003, Mann Whitney).

## Discussion

Simultaneous detection of 16S rRNA and IS*2404* by qPCR has been shown to be a specific marker for the presence of viable *M*. *ulcerans* in human tissue [15]. In this study, we have investigated the time taken for the 16S rRNA assay to become negative during antibiotic treatment for 8 weeks. The assay detected viable bacteria in 65% of samples taken from patients proven to have Buruli ulcer by PCR for IS*2404*. Since these samples were from untreated patients, they should all have been *M*. *ulcerans* 16S rRNA positive. One possible explanation for false negatives would be loss of mRNA during transport to the laboratory so we measured concurrent detection of human GAPDH mRNA. This was positive showing that mRNA was present in the 16S rRNA negative samples. Sampling error is the most likely explanation for the false negatives which is not surprising since the volume of FNA samples is less than 50 μl and *M*. *ulcerans* is not evenly distributed within lesions [19]. We found that there was a relationship between bacterial load measured by qPCR for IS*2404* and the result of the 16S rRNA assay; bacterial load was significantly lower in samples with negative 16S rRNA. Thus the combination of low bacterial load and a less sensitive 16S rRNA assay may also account for false negatives. The 16S rRNA assay was more sensitive than culture for *M*. *ulcerans* as shown in Table 2; negative 16S rRNA with positive culture was detected in only 2 patient lesions whereas negative culture with positive 16S rRNA was found in 48 lesions.

At week 4, 20 of 129 (16%) lesions had healed and 22% of unhealed lesions had no detectable viable *M*. *ulcerans* (16S rRNA) in the lesion (Fig 1). If these patients could be identified before or during the early stages of treatment it is possible that the course of antibiotics could be shortened substantially with considerable benefit to patients as well as a reduction in the cost of management. The recommendation that patients receive treatment for 8 weeks was derived from the finding that early lesions excised after 2 weeks antibiotic treatment were still culture positive but those excised after 4 weeks were all negative [3]. The 16S rRNA assay is more sensitive than culture as shown in the present study and if lesions could be shown to be 16S rRNA negative at 4 weeks it would be justified to abbreviate the course of antibiotics. This would need to be assessed by a clinical trial, using the currently recommended combination of clarithromycin and rifampicin. Evidence for shorter treatment for selected patients is supported by recent data from Australia where complete healing was achieved after 14 to 28 days of antibiotics in selected patients but most of the patients had received early surgical treatment in addition to antibiotics and the study was retrospective [8]. The cost and skill requirement for the 16S rRNA assay limits its routine use in most countries where Buruli ulcer is endemic but it may be possible to predict rapid responders in other ways. This is the subject of ongoing studies.

The healing rate was faster over the first 4 weeks in patients who had cleared active infection by that time (Fig 3). Also the time to complete healing was significantly longer in patients with persistent infection independently of the initial lesion size. There has been speculation about why some lesions heal slower than others despite appearing clinically comparable before treatment and the findings from this study suggest that persistent infection is an important contributing factor. Furthermore several observations imply that the initial bacterial load may determine the time to total clearance of viable bacteria from BU lesions. A crude estimate of bacterial load was made by quantifying the number of copies of IS*2404* using qPCR. A better estimate could be made by taking multiple samples or biopsies but this was not considered ethically acceptable. Given the limitations of the data it is not surprising that there was not a significant correlation between initial bacterial load and the time for which viable bacteria remained detectable but Fig 1 illustrates that they are probably related since the bacterial load in lesions with negative *M*. *ulcerans* 16S rRNA at week 0 was significantly lower than that in all other groups.

At the end of the standard 8 week period of antibiotic treatment 52% of lesions were 16S rRNA positive (Fig 1) raising the question whether antibiotic treatment should be prolonged for a selected subgroup of patients. We have found positive *M*. *ulcerans* culture in 2 patients who had fully complied with treatment for 8 weeks in an earlier study[12]. The finding that healing was delayed in this group compared with those with negative 16S rRNA supports the idea of continuing antibiotics, perhaps for a further 4 weeks but against this is the fact that all the lesions healed eventually without further antibiotic treatment. There is also the difficulty of identifying such lesions except within the context of a research study since this assay is relatively expensive and labor intensive for routine use. At present a judgment would have to be made on purely clinical grounds.

The presence of detectable *M*. *ulcerans* 16S rRNA after chemotherapy with rifampicin and streptomycin may be indicative sometimes of a persistent altered physiological state of *M*. *ulcerans* such that it can reactivate to cause recurrent disease later. An analogous situation arises when *M*. *tuberculosis* is treated with rifampicin or pyrazinamide. Subpopulations consisting of dormant or semi-dormant, antibiotic tolerant persisters survive longest during chemotherapy and are difficult to kill with any new antibacterial drug. They are thought to be responsible for the prolonged period required for effective chemotherapy in tuberculosis [20–22]. In human *M*. *ulcerans* disease, lesions with persistent viable organisms still go on to heal, albeit slowly, presumably due to immune clearance of the organism whereas in tuberculosis, residual viable organisms invariably cause disease. In BU, as mycolactone concentration decreases in lesions during antibiotic therapy [12], IFN-gamma levels [23] increase possibly due to *M*. *ulcerans* antigens interacting normally with the immune system. The slow clearance of these organisms may however explain the slow healing of some of these wounds due to the inhibition of vital wound healing factors by mycolactone.

It is not known whether antibiotic tolerant persisters cause relapse in *M*. *ulcerans* disease but current evidence does not support this. Recurrent *M*. *ulcerans* disease was fairly common before the antibiotic era when 6–47% of patients experienced relapse after surgical treatment alone, [24] [25] probably because there were residual *M*. *ulcerans* in apparently healthy tissue at resection margins [26]. However, since observed antibiotic therapy was introduced, reported series have shown relapse rates below 2% [7, 9]. Individuals with a deeply compromised immune system such as those co-infected with HIV are at risk of relapse or overwhelming disseminated disease but this is more likely due to the need for a competent immune response to clear infection [27] [28]. That the presence of *M*. *ulcerans* 16S rRNA indicates persistence of viable organisms in the tissue is supported by our previous findings that mycolactone can be detected in some patients after they finish antibiotics as can positive cultures for *M*. *ulcerans* [12]. The presence of mycolactone, the toxin secreted by *M*. *ulcerans*, probably indicates that viable organisms are still extant but the pharmacokinetics of mycolactone are not known and it could persist after killing of the organism. Mycolactone is a powerful inhibitor of many growth factors and if it persists in a Buruli ulcer it is likely to retard healing [29]. Further investigations are ongoing to identify lesions containing the toxin after the end of treatment in the present study. However further work is also needed to determine if there is an association between *M*. *ulcerans* 16S rRNA and mRNA detection suggestive of transcriptional activity which would indicate that the organisms are in a replicative state.

In conclusion this study has demonstrated that current antibiotic therapy for BU disease is highly successful in most patients but it may be possible to abbreviate the treatment to 4 weeks in patients with a low initial bacterial load. On the other hand evidence has been presented that persistent infection contributes to slow healing in other patients, probably those with a high bacterial load, who may need antibiotics for longer than 8 weeks.

## Supporting information

S1 Protocol Human GAPDH mRNA RT qPCR. (DOCX)

S2 Protocol Validation and performance of human GAPDH mRNA RT qPCR. (DOCX)

S1 Table Patient information. (XLSX)

S1 Checklist STROBE checklist. (DOCX)
