# Supplementary material for: Clearance of viable Mycobacterium ulcerans from Buruli ulcer lesions during antibiotic treatment as determined by combined 16S rRNA reverse transcriptase /IS 2404 qPCR assay
Source: PLoS Negl Trop Dis. 2017 Jul 3;11(7):e0005695. doi: 10.1371/journal.pntd.0005695 (PMC5510892; doi:10.1371/journal.pntd.0005695)
Supplement: S1 Protocol — (DOCX) [file pntd.0005695.s001.docx]

**Protocol S1.** Human GAPDH mRNA RT qPCR

Oligonucleotide sequences for primers were used as originally described by Janssens et al. [19]. The hydrolysis probe was modified with 6-FAM and BBQ for thermodynamic reasons and modifications of the reagents and run protocol were employed as described for 16S rRNA (RT)/IS*2404* qPCR assays.

Table S1.1 Primers and probe

| **Test** | **Primer/probe^a^** | **Sequence (5´- 3´)^b^** | **Nucleotide Position^c^** | **Amplicon Size** |
| --- | --- | --- | --- | --- |
| GAPDH mRNA  RT qPCR | GAPDH fwd | GAA GGT GAA GGT CGG AGT C | 180 - 189 | 225 bp |
|  | GAPDH rev | GAA GAT GGT GAT GGG ATT TC | 386- 405 |  |
|  | GAPDH TM | FAM-CAA GCT TCC CGT TCT CAG CCT -BBQ | 356- 376 |  |

^a^Hydrolysis probe with 6 FAM, 6-Caboxyfluorescein fluorescent dye and BlackBerry Quencher (BBQ)

^b^Nucleotide positions are provided for the sequence in *Homo sapiens* glyceraldehyde-3-phosphate dehydrogenase (GenBank accession number: NM_002046.5)

The reaction mixture contained 1 µl of 10µM each of primers GAPDH forward, GAPDH reverse and probe GAPDH TM, 4 µl of 5x HOT FIREPol Probe qPCR plus, 2 µl of exogenous IPC reagent and 0.4 µl of exogenous IPC DNA.

Amplification of GAPDH (cDNA) targets was carried out at 95^o^C for 15 min, and then 40 cycles of 95^o^C for 15 sec and 60^o^C for 60 sec in a BioRad CFX 96 real-time PCR detection system.
