# Supplementary material for: Clearance of viable Mycobacterium ulcerans from Buruli ulcer lesions during antibiotic treatment as determined by combined 16S rRNA reverse transcriptase /IS 2404 qPCR assay
Source: PLoS Negl Trop Dis. 2017 Jul 3;11(7):e0005695. doi: 10.1371/journal.pntd.0005695 (PMC5510892; doi:10.1371/journal.pntd.0005695)
Supplement: S2 Protocol — (DOCX) [file pntd.0005695.s002.docx]

**Protocol S2**. Validation and performance of human GAPDH mRNA RT qPCR

Establishment and technical validation of the GAPDH mRNA reverse transcriptase qPCR assay was performed at DITM.

Specificity of the assay was assessed in silico using the basic local alignment search tool (BLAST, GenBank, NCBI) and in vitro by testing “must detect GAPDH” samples and “must not detect GAPDH” samples as indicated in table S2.1. The assay was 100% specific for the human GAPDH mRNA.

Table S2.1. Samples used for technical validation

| **Purpose** | **Sample type (amount)** | **Obtained from** | **Origin** |
| --- | --- | --- | --- |
| **"must detect GAPDH" samples** | Nasal swab (7)^a,b^ | 7 clinically diagnosed MB leprosy patients (laboratory confirmation by RLEP qPCR) | Togo |
|  | Skin biopsy (3) ^a, b^ | 3 clinically diagnosed Onchocerciasis patients (laboratory confirmation by O-150 qPCR) | Ethiopia |
|  | PCR standard | Cloned GAPDH plasmids with known copy numbers | GenExpress, Berlin, Germany |
| **"must not detect GAPDH" samples** | Mycobacterial culture (2) ^a^ | *M. ulcerans* (2 different strains) | Cameroon |
|  | Mycobacterial culture (13)^a^ | *M. chelonae, M. fortuitum, M. gordonae, M. intracellulare, M. kansasii, M. lentiflavum, M. malmoense, M. marinum, M. scrofulaceum, M. smegmatis, M. szulgai, M. tuberculosis, M. xenopi* | National Reference Center for Mycobacteria, Borstel, Germany |
|  | Mycobacterial culture (6)^a^ | *M. gordonae, M. kansasii, M. lentiflavum, M. marinum, M. tuberculosis, M. xenopi* | Bernhard-Nocht Institute, Hamburg, Germany |

^a^DNA extract

^b^These “must detect GAPDH” sample extracts include human cell material and therefore also human GAPDH DNA which has the same nucleotide sequence in this assay as cDNA generated from human RNA.

Sensitivity was assessed by testing the above mentioned “must detect GAPDH” samples and was 100%.

To exclude false negative results in different matrices of clinical samples, aliquots of ten “must not detect GAPDH” samples were spiked with GAPDH plasmid DNA, subjected to GAPDH qPCR and all tested positive.

The intra-assay variability was evaluated by testing each sample from the respective logarithmic dilution in duplicate within one 96-well qPCR plate in one run. Inter-assay variability was assessed by testing each sample on two subsequent days. The concordance rate of positive and negative results was 100%.

To assess qPCR efficiency, a standard curve was generated by means of 4 logarithmic dilutions of the plasmid standards (10^8^-10^5^). The efficiency (E) was calculated using the slope of the regression line (y) of the standard curve with E=10^-1/y^-1. E values >0.95 were considered acceptable. The efficiency E was 100% with R^2=0,999 and a slope of -3,302 (figure S2.1).


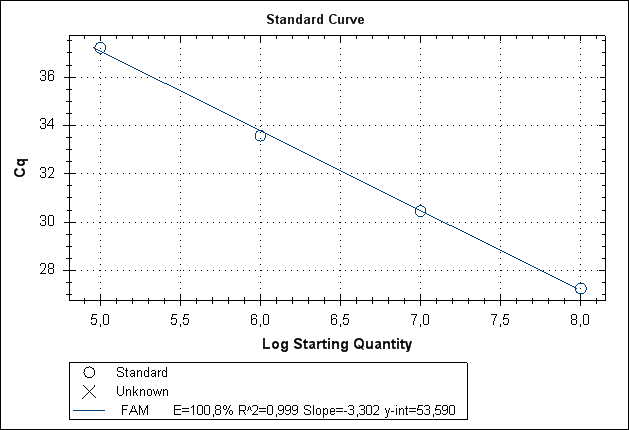


Figure S2.1 Standard curve for the GAPDH mRNA RT qPCR assay

The lower limit of detection (LOD, i.e. lowest template concentration rendering >95% positive results) was assessed by serial dilutions of plasmid standards. The LOD was 100 copies of the target sequence.
